# Quantitative trait loci mapping of resistance to pre-harvest sprouting in the Norwegian spring wheat breeding line T7347

**DOI:** 10.1007/s00122-025-04943-7

**Published:** 2025-06-24

**Authors:** Anja Karine Ruud, Most Champa Begum, Anne Kjersti Uhlen, Ennian Yang, Min Lin, Ellen Færgestad Mosleth, Jon Arne Dieseth, Morten Lillemo

**Affiliations:** 1 Department of Plant Sciences, Norwegian University of Life Sciences, Post Box 5003, 1432 Ås, Norway; 2 Crop Research Institute, Sichuan Academy of Agricultural Sciences, Chengdu, 610066 Sichuan China; 3 Nofima AS, Osloveien 1, 1433 Ås, Norway; 4 Graminor, Hommelstadvegen 60, 2322 Ridabu, Norway

## Abstract

****Key message**:**

**QTL mapping of the Saar x T7347 RIL population identified five stable pre-harvest sprouting QTL. Effects of four of the QTL were validated in modern breeding line populations.**

**Abstract:**

Pre-harvest sprouting (PHS) can result in downgrading of food quality wheat to feed in seasons with high humidity and precipitation before harvest. Higher temperatures during the grain filling phase further reduce the dormancy level and increase the risk of PHS. However, some genotypes, including the Norwegian breeding line T7347, show a high level of dormancy even under elevated temperatures. In this study, the main objective was to investigate the genetic mechanisms behind the high dormancy in T7347. A population of 233 recombinant inbred lines was developed by crossing T7347 with Saar, a CIMMYT line with moderate to low level of dormancy. The population was grown in a total of 13 field trials at three different locations in Norway and Chengdu, China, and screened for germination index (GI) and falling number (FN). The population was genotyped with the TraitGenetics 25 K SNP chip, and QTL interval mapping revealed five stable PHS QTL on chromosomes 1A, 3A, 3B, 7A and 7B. Among these, the largest proportion of phenotypic variation of GI and FN was explained by QTL overlapping with the known red color loci on chromosomes 3AL and 3BL, with the alleles conferring dormancy contributed by T7347 and Saar, respectively. Additionally, two unique FN QTL were identified, on 4B overlapping with semi-dwarfing gene *Rht-B1*, and on 5AL co-located with the *Tipped 1* awn locus. The effect of four of the PHS QTL and the two FN QTL could be validated in independent panels of advanced breeding lines.

**Supplementary Information:**

The online version contains supplementary material available at 10.1007/s00122-025-04943-7.

## Introduction

Pre-harvest sprouting (PHS) is the germination of seeds on the mother plant before harvest. Selective sowing and harvesting during the domestication process of wheat lead to a decrease in seed dormancy (Liu et al. 2015), facilitating more uniform germination and immediate replanting after harvest, but also an increased risk of PHS under unfavorable environmental conditions. The risk of PHS increases with high relative humidity or rain. Seed dormancy builds up during grain maturation (Chahtane et al. 2016), and higher temperatures in this period generally reduce the final level of dormancy (Yan and Chen 2020). When germination is initiated in a cereal crop such as wheat and hydrolytic enzymes, including *α*-amylases and proteases degrade starch and proteins in the endosperm to provide energy, leading to reduced yield (test weight) and quality (Ross and Bettge 2009). Starch degradation leads to reduced gelling capacity of the flour, low falling number (FN) and loss of baking quality (Derera 1989). Therefore, affected lots may be degraded from food to feed. Sprouted grain has higher activity of oxidoreductases and hydrolases, which increases the risk of spoiling and lower nutrient levels (Li et al. 2004). Increased and premature *α*-amylase activity can also be caused by late maturity *α*-amylase (LMA), which is induced by temperature shock during grain maturation (Derkx and Mares 2020; Lunn et al. 2001; Mares and Mrva 2014). Under most conditions, the synthesis of *α-*amylase occurs during early stages of maturation. Later, new synthesis usually only happens in ripe/near-ripe grains after physiological (yellow) ripeness if germination is triggered by wetting (Mares and Mrva 2008). In LMA-affected grain, some studies indicate that new synthesis of high pI (isoelectric point) *α-*amylase occurs in the aleurone at the later stages of maturation and does not require wetting as reviewed by Mares and Mrva (2008). Premature synthesis of *α*-amylase can be inhibited by high temperatures during grain filling in gibberellin (GA)-insensitive genotypes, namely, those harboring semi-dwarfing alleles *Rht-B1b* or *Rht-D1b* (Mrva and Mares 1996). On the other hand, wild-type tall varieties express LMA under a broader range of temperatures, and LMA activity can be induced by temperature shock in GA insensitive genotypes (Derkx et al. 2021; Derkx and Mares 2020; Mrva and Mares 2001).

Quantitative trait loci (QTL) for PHS have been identified on all wheat chromosomes, as reviewed by Tai et al. (2021). Among the many QTL, several have been consistently detected in different mapping populations and environments. Although the gene function has not always been identified, some genes have been more extensively studied, as reviewed by Vetch et al. (2019).

Red wheats are generally more resistant to PHS than white wheats (Himi et al. 2002). The pigment of the red-grained pericarp consists of flavonoids, i.e., catechin and proanthocyanidins (PAs) (Miyamoto and Everson 1958; McCallum and Walker 1990), and the pericarp color is controlled by three homologous genes *Red-A1 (R-A1)*, *R-B1* and *R-D1* on the group 3 chromosomes. These *R* genes, which encode MYB10 transcription factors, are involved in both ABA signal transduction and the flavonoid synthesis pathway (Himi et al. 2011, 2002). The phytohormones absisic acid (ABA) and gibberellin (GA) act antagonistically in signal transduction as the primary regulators of seed dormancy and early germination (Tuan et al. 2018). A higher ratio of ABA to GA increases dormancy levels, while a higher relative ratio of GA promotes germination (Toora et al. 2024). However, studies have also reported no significant differences in concentration of these hormones between dormant and non-dormant lines and have suggested that differences in sensitivity rather than concentration are important (Himi et al. 2002, 2024; Matsuura et al. 2019). Reactive oxygen species (ROS), including H_2_O_2_, cause oxidative damage to lipids, proteins and nucleic acids, but have also been proposed as signaling molecules and regulators of several processes including hormone signaling and dormancy control (El-Maarouf-Bouteau and Bailly 2008; Mittler et al. 2004). H_2_O_2_ has been documented to inactivate enzymes involved in ABA signaling (Meinhard and Grill 2001; Meinhard et al. 2002). Plant metabolites with antioxidant properties, such as PAs, can inhibit ROS factors and thus play an important role in the redox regulation of dormancy (Bailly et. al. 2008). The *R* genes are considered additive in terms of their contribution to increased dormancy (Flintham 2000). However, the largest difference in dormancy is observed when comparing white genotypes with genotypes with one or more *R* genes. When comparing 73 red genotypes derived from a cross between Timgalen (white) and RI4137 (with all three *R* genes) with different number of *R* genes, the effect of increased *R* dosage was associated with small increases in sprouting resistance, significant for a 3-year average, but only in one out of three individual trial years (Bassoi and Flintham 2005). The same study did not find significant correlation between *R* gene dosage and grain color intensity recorded by visual assessment of grain treated with sodium hydroxide (NaOH) to enhance the color, but identified similar correlations between color intensity and sprouting as for R gene dosage and sprouting. In addition to visual inspection of the NaOH-treated seeds, the reflectance spectra captured by NIR/VIS instruments can also be used to assess the seed coat color (McCaig et al. 1993; Wang et al. 1999).

PHS is a complex trait affected by environmental factors and other genetic mechanisms than the above-mentioned plant hormones. Other mechanisms involve vegetative and reproductive components of spike morphology including awns, germination inhibitors in the bracts and opening of florets (Imtiaz et al. 2008). For example, genotypes with awns have been shown to absorb approximately 30% more water into the spikelet than awnless genotypes (King and Richards, 1984). Once the grain reaches critical water content, sprouting will occur and awnless spike morphology can delay pre-harvest sprouting compared to awned morphology under humid conditions before harvest.

PHS resistance is usually assessed by germination index (GI) (Hagemann and Ciha 1984; Reddy et al. 1985) or sprouting score (McMaster and Derera 1976) or indirectly with Hagberg falling number (FN) (Hagberg 1961). FN is a measure of wheat quality and determines the level of starch degradation in the flour by measuring the time it takes for a stirring paddle to drop through a mix of whole meal flour and water. However, a decreased FN is not ONLY always linked to PHS, but also to additional causes, including LMA as described above.

Temperature is one of the environmental factors influencing dormancy during seed development and germination. Low temperature during grain development increases the level of dormancy (Buraas and Skinnes 1985; Reddy et al. 1985). One gene shown to be involved of controlling the temperature response to dormancy is a wheat *MOTHER OF FT AND TFL1* homolog on the short arm of chromosome 3A (*MFT-A1*) (Nakamura et al. 2011). In this study, we screened a biparental population of 233 recombinant inbred lines (RILs) for segregation of PHS resistance assessed by germination index (GI) and Hagberg’s falling number (FN). Among the parents, the Norwegian breeding line T7347 was selected because it maintains a high level of dormancy even under elevated temperatures (Buraas and Skinnes 1985). In her recent PhD thesis, Begum (2023) compared GI for T7347 and Saar grown under different temperature regimes (17/12, 22/17 and 27/22 °C day/night) and showed that while GI for T7347 only increased from approximately 0.1 at the lowest temperature to just below 0.3 at the highest, Saar increased from approximately 0.3 to 0.5. Since Saar has a relatively low level of dormancy and PHS resistance, the offspring of the cross between T7347 and Saar were expected to segregate for FN and GI.

## Materials and methods

### Plant material and field trials

The recombinant inbred line (RIL) Saar x T7347 (SxT) population was generated from a cross between the CIMMYT spring wheat variety Saar, which has a low level of dormancy, and the Norwegian breeding line T7347, selected for its strong level of dormancy (Buraas and Skinnes 1985). Saar is awned, while T7347 is awnless. The cross was performed both ways, and the population consisted of a balanced number of offspring with Saar as a father (125) and mother (126), respectively. From the F2 population, single seed descent was performed until F6 when DNA was extracted. Seedlings were grown in the greenhouse, and genomic DNA was extracted from fresh young leaves using the DNeasy plant DNA extraction kit (Qiagen). The parents and 251 RILs were genotyped with the TraitGenetics Illumina 25 K SNP Chip (SGS Institut Fresenius – TraitGenetics section, Gatersleben, Germany). The F6 RILs and parents were grown at two locations in south-eastern Norway, Vollebekk research station and Staur in 2018, 2019, 2021 and 2022, and in Chengdu, China, in 2020 and 2021.

At Vollebekk, two field trials were sown each year. One trial was used to assess GI and harvested for FN measurements right after maturity was reached for all lines. The second trial was subjected to delayed harvest, and in case of lack of natural rainfall, mist irrigation was applied from the end of the grain filling period until the PHS susceptible parent Saar had reached FN below 100. The mist irrigation scheme involved misting for 30 min four times in two consecutive afternoons/evenings twice per week. The amount of water applied on the days of mist irrigation was measured to approximate 10 mm of precipitation. Trials subjected to mist irrigation are identified with “mist” in the trial names.

Additionally, FN data were collected from validation panels consisting of advanced breeding lines of spring wheats from Graminor AS’s breeding program, grown in six trials in Vollebekk, Rød, Staur and Bjørke between 2019 and 2022. These populations varied in size and composition between years (see Table S1 for overview) and were genotyped with the same 25 K SNP Chip. The trials at Vollebekk were supplemented with mist irrigation as described above.

### Phenotype data

The trials were sown in the autumn in China and in the spring in Norway. The plants were scored for heading date, plant height and yellow maturity to allow for the estimation of confounding effects or pleiotropy. Additionally, the awn presence/absence in the RILs was scored after heading in the 2022 FN trial in Vollebekk.

For GI assessment in Norway (Vollebekk 2018–2021), 10 spikes per plot were sampled at yellow maturity. The method is described in more detail in Begum et al. (2024). Briefly, the spikes were dried at 25 °C in drying chambers for 72 h to reduce water content below 15% and preserved at -20 °C to prevent the release of dormancy (Lin et al. 2016). Immediately before the GI tests were performed, the spikes were removed from the freezer and threshed using a threshing machine. Germination tests were cconducted on petri dishes with wetted filter paper at 19 °C. Seeds were counted daily as germinated when radicles were visible after the emergence from the embryo, and germinated seeds were then removed from the petri dish. The analyses were performed in duplicates of 25 seeds for each sample. GI was calculated using following formula described by Walker-Simmons (1987) with a slight modification: $GI=(14\times n1+13\times n2+\dots +1\times n14)/(14\times N-M)$, where *n1*, *n2*, …*n14* are the number of seeds germinated on the first, second, and *n*th days until the 14th day, respectively; *N* is the total number of seeds placed on the petri dish and *M* is the total number of seeds that had been removed during the 14 days incubation period due to infestations with molds.

From the field trials conducted in Chengdu, the spikes for GI analyses were harvested at the same date for all the lines around maturity. The spikes were threshed and stored in the freezer at -20 °C. Fifty well-developed seeds from each line were incubated for germination test at 20 °C for 7 days. Two technical replications were performed for each sample, and GI was calculated by following Walker-Simmons (1987) formula with the modifications as explained above.

For FN analysis, individual plots from the field trials in Norway were harvested as normal and then dried in a drying chamber with ventilator at room temperature to reduce moisture content below 15%. Grains were cleaned and milled with a Perten 3100 FN mill (Perten Instruments AB, Huddinge, Sweden). For the FN test, 7 g of whole meal flour with moisture content of 15% was mixed with 25 mL of distilled water and analyzed according to Perten (2005). Two technical replications were performed at a time for each sample to calculate mean FN. Samples with differences of more than 20 s between replications were repeated.

Whole grain kernels of 233 RILs and parents from the 2018 field trial in Vollebekk were scanned by NIR/VIS (near-infrared–visible) reflectance using FOSS NIR Systems XDS Rapid Content Analyzer (FOSS Analytics A/S, Hillerød, Denmark). The measurements were repeated on three seed samples per genotype. The reflectance values for the VIS (visible) part of the spectra at wavelength 400–800 nm were normalized with Standard Normal Variate (SNV) pre-processing. SNV performs a normalization of the data by subtracting each spectrum by its own mean and dividing it on its own standard deviation (Barnes et al. 1989). PCA was performed on the reflectance values for the VIS spectra (400–800 nm), and PC1 eigenvectors used as a trait for QTL mapping. The visible part of the spectrum was used since this is the part of the light spectrum perceived as color to the human eye.

### Broad sense heritability

Broad sense heritabilities were calculated separately for all trials for GI and FN, and across trials for GI and FN within Norway, with the equation$$H^{2} = ~{\raise0.7ex\hbox{${\sigma _{g}^{2} }$} \!\mathord{\left/ {\vphantom {{\sigma _{g}^{2} } {(\sigma _{g}^{2} + ~\sigma _{E}^{2} /t + ~\sigma _{e}^{2} /\left( {t*r} \right))~~}}}\right.\kern-\nulldelimiterspace} \!\lower0.7ex\hbox{${(\sigma _{g}^{2} + ~\sigma _{E}^{2} /t + ~\sigma _{e}^{2} /\left( {t*r} \right))~~}$}}$$, where $${\sigma }_{g}^{2}$$ is the genetic variance, $${\sigma }_{E}^{2}$$ is the environmental variance, $${\sigma }_{e}^{2}$$ is the error variance, *t* is the number of trials and *r* is the number of replicates per trial.

### Genetic linkage map construction and QTL mapping

For map construction in JoinMap 4 (van Ooijen 2006), we excluded markers and RILs with > 0.1 heterozygosity, > 0.2 missing data and MAF < 0.2, as well as RILs with more than 95% similarity to a parent. Only markers segregating between the parents were kept.

In total, 233 RILs were kept, and 8625 SNP markers were used as input for JoinMap. Prior to map construction, explorative genome-wide association mapping (GWAS) was done by running MLM in GAPIT (Wang and Zhang 2021) using the physical map positions [RefSeq_v.1.0 (IWGSC, 2018)] and phenotype data to identify chromosome regions associated with differences in GI or FN. We used these results from GWAS to prioritize linkage groups to include in the map. The genetic distances between markers were calculated in JoinMap by converting recombination fractions into map distances (centiMorgan, cM) based on the Haldane’s mapping function which is always used for the maximum likelihood mapping algorithm with minimum LOD score of 3.0 (Haldane 1919).

QTL mapping was done with MapQTL6 (van Ooijen 2009). Both interval mapping (IM) and multiple QTL mapping (MQM) were performed. The LOD significance threshold was set to 3.0. MapChart, v.2.32 (Voorrips 2002) was used to draw the genetic maps and LOD curves. Unmapped markers were also included for interval mapping in MapQTL to increase the chances of capturing markers associated with the traits.

After the preliminary analysis showed QTL peaks in regions on chromosomes 3AL and 3BL overlapping with the *TaMyb10-A1* and *TaMyb10-B1* transcription factors affecting red grain color in wheat, 199 RILs and the parents were genotyped with gene-specific KASP markers targeting the homeologues on 3A, 3B and 3D.

### QTL effects

The most significant markers linked to the PHS QTL (QTL with effect on both GI and FN, Table S1) were used for allele stacking in the SxT RIL population, and Tukey’s honest significance differences (HSD) test was used to test the effects on FN and GI between RILs harboring different numbers of QTL alleles. To test whether the QTL identified in the SxT population were relevant in modern breeding material adapted to Norwegian conditions, the effects of markers linked with the GI and FN-related QTL were tested in advanced breeding line panels, using a paired t-test for a total of 6 trials conducted in different locations within south-eastern Norway (Staur, Bjørke, Rød, Vollebekk, Fig. S4, Table S2). The breeding line panels varied in number of genotypes between years (Table S2). Since these lines were not genotyped with the gene-specific *TaMyb10*-markers, we used the closely linked SNP markers on 3A and 3B (Table S3).

## Results

### Phenotype data

**Fig. 1:**
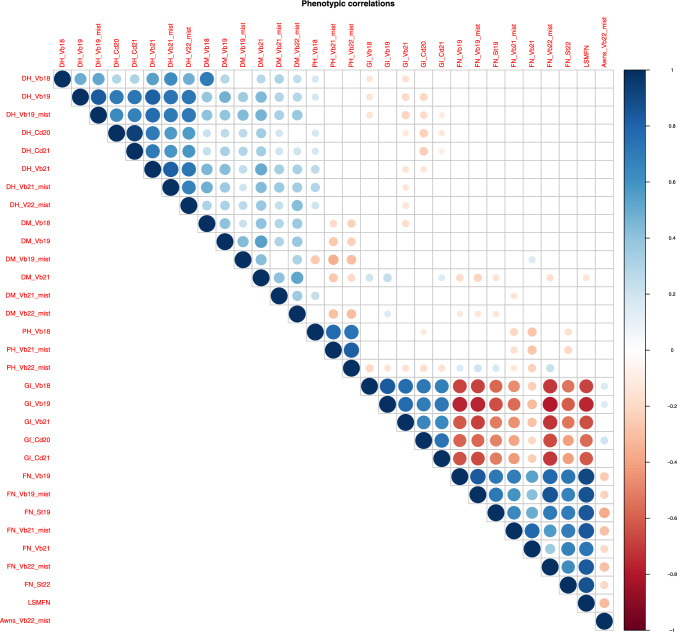
Correlation plot between phenotypes from the field trials in Vollebekk (Vb), Staur (St) and Chengdu (Cd), DH = days to heading, DM = days to maturity, PH = plant height, GI = germination index, FN = falling number and LSMFN = LS means of FN data across trials. Only significant correlations (*p* ≤ 0.05) are shown as circles in the plot

In total, we used FN data collected from seven experiments in Vollebekk and Staur (2019, 2021 and 2022), and GI data from five experiments in Vollebekk (2018, 2019 and 2021) and Chengdu (2020 and 2021) (Fig. 1, Figure S1). Additionally, agronomical data such as days from sowing to heading (DH) and days from sowing to maturity (DM) were available from most of the trials, plant height (PH) from three trials (2019, 2021 and 2022) and awn presence/absence in one (2022) (Fig. 1). Significant correlation between plant height to both GI and FN was observed, as well as significant negative correlation between days to heading and GI trials and between DM in 2021 and FN.

The phenotype histograms of Least Squares (LSmeans) (Figure S1) indicated that most phenotypes were quantitative. However, the Shapiro–Wilk test (Shapiro and Wilk 1965) (results not shown) revealed that normal distribution was not achieved for many traits and trials (none of the GI trials). Figure S4 shows the phenotypic distribution of FN for the independent advanced breeding line validation panels, and Table S1 provides an overview of the number of phenotyped and genotyped lines in these trials.

Grain color was assessed as the reflectance values from the VIS spectra, and PCA analysis was performed on these values. PC1 explained 92.2% (Figure S2 and Fig. 3) of the phenotypic variation between the RILs and was used as an input trait to map grain color in the QTL analysis (Table 2). The histogram of the PC1 eigenvectors showed a left-skewed distribution where the parents were relatively similar in value (Figure S3), indicating that they had similar color.

### Broad sense heritability

The correlations between agronomical traits days to heading (DH) and days to maturity (DM) to germination index (GI) varied between trials but were negative and significant for all trials except Vollebekk 2019 (Fig. 1), and most significant for the trial in Chengdu, China, in 2020. Generally, the correlation between plant height (PH) and FN was insignificant, with the trials in Vollebekk 2021 as an exception where FN and PH were negatively correlated. Additionally, a negative correlation (p < 0.05) was observed between PH (measured in Vollebekk 2018 and 2021) and GI in China 2020 (Fig. 1).

Broad sense heritability, *H*^*2*^, was high for FN and GI within and between trials (Table 1). This is in accordance with other studies (Imtiaz et al. 2008; White et al. 2022). Heritabilities were not calculated for the trials in Chengdu since they were not replicated.

**Table 1:** Broad sense heritabilities *(H*^*2*^*)* within and between trials for falling number (FN) and germination index (GI)

Trial	*H*^*2*^
Vb_FN_2019_mist	0.89
St_FN_2019	0.92
Vb_FN_2019	0.91
Vb_FN_2021 _mist	0.84
Vb_FN_2021	0.89
Vb_FN_2022_mist	0.91
St_FN_22	0.94
Vb_GI_2018	0.87
Vb_GI_2019	0.89
Vb_GI_2021	0.89
GI across (within Norway)	0.85
FN across (within Norway)	0.82

Locations: Vb = Vollebekk, Norway, St = Staur, Norway, Cd = Chengdu, China

### QTL mapping

QTL were reported here if they were significant (LOD above 3) in more than one trial, and defined as PHS QTL if they were significant for both GI and FN.

**Fig. 2:**
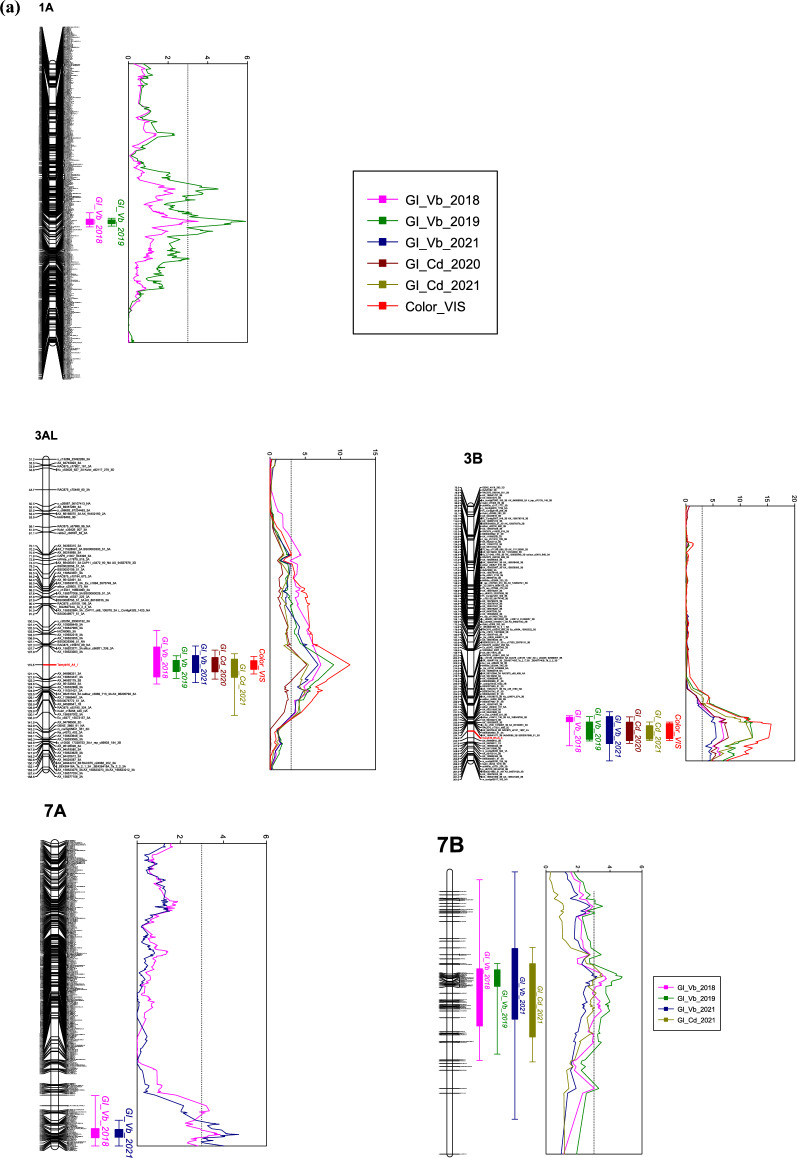

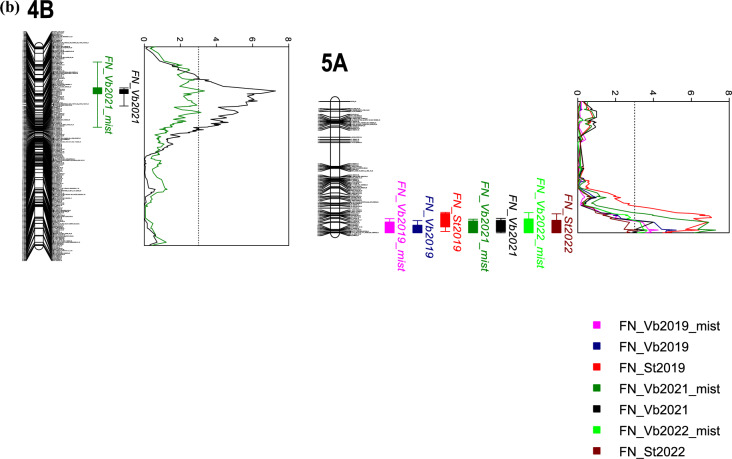
**a** Linkage maps with the five PHS QTL. LOD curves from IM in MapQTL were based on GI and VIS phenotype, and LOD curves for FN are not shown to avoid cluttering but overlap with the presented QTL. The red LOD curve on chromosomes 3AL and 3BL represents PC1 for VIS results (grain color), and the *TaMyb10-A1* and *TaMyb10-B1* markers are marked in red on the linkage groups, and **b** linkage maps with the FN-specific QTL. LOD curves on 4B and 5A from IM in MapQTL

The PHS QTL explaining the largest percentage of the phenotypic variance in most environments were located on the long arms of 3AL and 3BL, the QTL on 3B explaining up to 20.3% of the phenotypic variation for FN and 21.5% of the phenotypic variation for GI (Table 2). Using the PC1 scores of the VIS data identified QTL for red seed color in the same QTL regions on 3A and 3B (Table 2 and Fig. 2a). The most significant marker on 3A was the *TaMyb10-A1* KASP marker, located at 703.905 Mbp. On chromosome 3B, the *TaMyb10-B1* marker in position 757.92 Mbp was only the most significant marker in the 2020 mist irrigated FN trial in Vollebekk. However, the peak was always located between 231 and 244 cM and 749–759 Mbp on 3BL, indicating that either the *TaMyb-3B1* was underlying the QTL, or there may be other closely linked genes.

**Table 2:** QTL for GI and FN in Saar x T7347 were reported here if they were detected in at least two trials/environments

Chr./QTL name	cM pos	Mbp pos	Peak marker	LOD	%PEV	Beneficial allele	Trial^1^	Comment
1A	147.670	460.33	*AX-158537396*	3.52	6.7	T7347	GI2018_Vb	
*QPHS.nmbu.1A.1*	147.670	460.33	*AX-158537396*	5.92	11.1	T7347	GI2019_Vb	
	147.221	458.40	*tplb0046g22_1351*	3.11	6	T7347	FN2019_Vb_mist	
	147.221	458.40	*tplb0046g22_1351*	4.4	8.3	T7347	FN2019_St	
	147.670	460.33	*AX-158537396*	4.07	7.7	T7347	FN2022_Vb_mist	
	147.221	458.40	*tplb0046g22_1351*	3.1	6	T7347	FN2022_St	
	147.221	458.40	*tplb0046g22_1351*	3.62	6.9	T7347	LSM_FN	
3A	64.438	703.905	*TaMyb10-3A1*	7.23	13.3	T7347	GI2018_Vb	
*QPHS.nmbu.3A.1*	64.438	703.905	*TaMyb10-3A1*	9.06	16.4	T7347	GI2019_Vb	
	64.438	703.905	*TaMyb10-3A1*	6.85	12.7	T7347	GI2021_Vb	
	64.438	703.905	*TaMyb10-3A1*	5.21	9.8	T7347	GI2020_Cd	
	64.438	703.905	*TaMyb10-3A1*	5.49	10.3	T7347	GI2021_Cd	
	64.438	703.905	*TaMyb10-3A1*	11.61	20.5	T7347	FN2019_Vb	
	64.438	703.905	*TaMyb10-3A1*	7.98	14.6	T7347	FN2019_Vb_mist	
	64.438	703.905	*TaMyb10-3A1*	9.19	16.6	T7347	FN2022_Vb_mist	
	64.438	703.905	*TaMyb10-3A1*	5.29	9.9	T7347	FN2022_St	
	64.438	703.905	*TaMyb10-3A1*	8.1	14.8	T7347	LSM_FN	
	64.438	703.905	*TaMyb10-3A1*	11.37	21.6	T7347	VIS_PC1	
3B	231.190	750.901*	AX-94481626*	7.06	13	Saar	GI2018_Vb	*Best BLAST hit on 3B
*QPHS.nmbu.3B.1*	244.688	757.932	AX-94896615*	12.26	21.5	Saar	GI2019_Vb	
	244.688	757.932	AX-94896615*	5.57	10.4	Saar	GI2021_Vb	
	244.688	757.932	AX-94896615*	7.81	14.3	Saar	GI2020_Cd	
	244.688	757.932	AX-94896615*	12.22	21.5	Saar	GI2021_Cd	
	233.593	754.97*	AX-95165551*	8.56	15.6	Saar	FN2019_Vb	
	233.593	754.97*	AX-95165551*	11.47	20.3	Saar	FN2019_Vb_mist	
	229.942	749.555	*BS00077967_51*	6.17	11.5	Saar	FN2019_St	
	239.758	757.92	*TaMyb10-3B1*	10.81	19.2	Saar	FN2022_Vb_mist	
	233.593	754.97*	AX-95165551*	6.32	11.7	Saar	LSM_FN	
	240.204	759.169	*IAAV6088*	15.67	28.5	Saar	VIS_PC1	
7A	187.871	712.059	*Ku_c19745_892*	3.86	7.4	T7347	GI2018_Vb	
*QPHS.nmbu.7A.1*	187.871	712.059	*Ku_c19745_892*	4.71	8.9	T7347	GI2021_Vb	
	173.531	701.294	*Excalibur_rep_c102327_102*	3.31	6.3	T7347	FN2022_Vb_mist	
7B	47.938	203.029	*Tplb0022m23_742*	3.79	7.2	T7347	GI2018_Vb	
*QPHS.nmbu.7B.1*	47.493	221.273*	*Tdurum_contig25631_143*	4.75	9	T7347	GI2019_Vb	*most significant blast hit on 7B
	47.493	221.273*	*Tdurum_contig25631_143*	3.18	6.1	T7347	GI2021_Vb	
	47.493	221.273*	*Tdurum_contig25631_143*	3.53	6.7	T7347	GI2021_Cd	
	47.493	221.273*	*Tdurum_contig25631_143*	3.7	7.1	T7347	FN2019_Vb_mist	
	47.493	221.273*	*Tdurum_contig25631_143*	4.88	9.2	T7347	FN2022_Vb_mist	
	47.938	203.029	*Tplb0022m23_742*	3.42	6.5	T7347	FN2022_St	
	47.493	221.273*	*Tdurum_contig25631_143*	4.2	8	T7347	LSM_FN	
Chr./QTL	cM pos	Mbp pos	Peak marker	LOD	%PEV	Beneficial allele	Trial^1^	Comment
4B	144.18	30.86	*TG0010a*	3.34	6.4	Saar	FN2021_Vb_mist	
*QFN.nmbu.4B.1*	144.18	30.86	*TG0010a*	7.27	13.4	Saar	FN2021_Vb	
5A	103.492	706.541*	*RAC875_c48885_142*	5.2	9.8	T7347	FN2019_Vb	*most significant blast hit on 5A
*QFN.nmbu.5A.1*	103.492	706.541*	*RAC875_c48885_142*	4.25	8.1	T7347	FN2019_Vb	
	93.402	698.51*	*RAC875_c8642_231*	7.07	13	T7347	FN2019_St	
	103.492	706.541*	*RAC875_c48885_142*	7.29	13.4	T7347	FN2021_Vb_mist	
	103.714	706.43	*AX-158538892*	3.67	7	T7347	FN2022_Vb_mist	
	103.492	706.541*	*RAC875_c48885_142*	3.17	6.1	T7347	FN2022_St	
	103.492	706.541*	*RAC875_c48885_142*	6.83	12.6	T7347	LSM_FN	

QTL are named PHS if they were significant for both GI and FN. The trials were conducted between 2018 and 22 (Vb = Vollebekk, St = Staur and Cd = China). Some FN trials were mist-irrigated as indicated with “mist” in the trial names. Peak markers from interval mapping (IM) are reported. The parental source of dormancy or high falling number is reported in the “Beneficial allele” column. % PEV = Percent phenotypic explained variance. LSmeans for FN were calculated across seven experiments in Norway. QTL for PC1 scores based on VIS reflectance values are also included

Trial abbreviations: *Vb* Vollebekk, Norway, *St* Staur, Norway, *Cd* Chengdu, China, and *Mist* mist irrigated trial

QTL on 1A, 7A and 7B were also important in several trials (Table 2, Fig. 2a). Additionally, two unique FN QTL (Table 2, Fig. 2b) were located on 4B and 5A.

### QTL effects

#### Effects of number of grain color genes

**Fig. 3:**
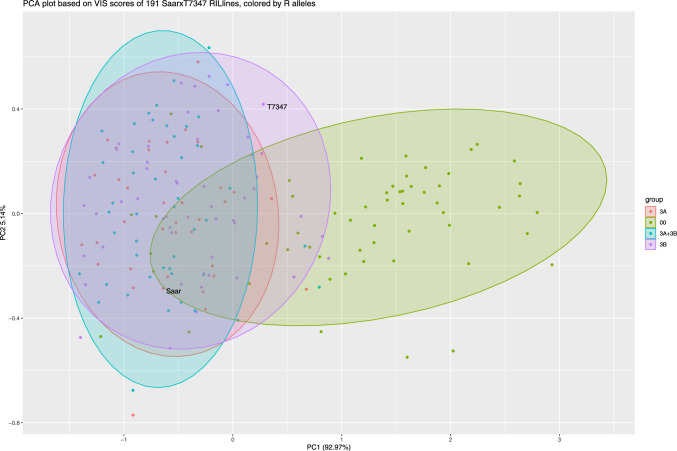
PCA plot showing PC1 and PC2 based on reflectance values in the visible spectrum (for all wavelengths 400–800 nm) of the grain for 191 RILs plus the parents Saar and T7347. The genotypes segregated for *TaMyb10-A1* and *TaMyb10-B1* and are colored by which allele they harbor based on the gene-specific KASP markers

The PCA plot in Fig. 3 showed how the RILs and the parents Saar and T7347 clustered based on PC scores calculated from the normalized VIS reflectance values. The genotypes were colored by which R-allele combination they harbored, and the results indicate that PC1 and PC2 could separate most RILs with no R gene (in red) from RILs with color genes, but that RILs with one or two genes clustered together.

**Fig. 4:**
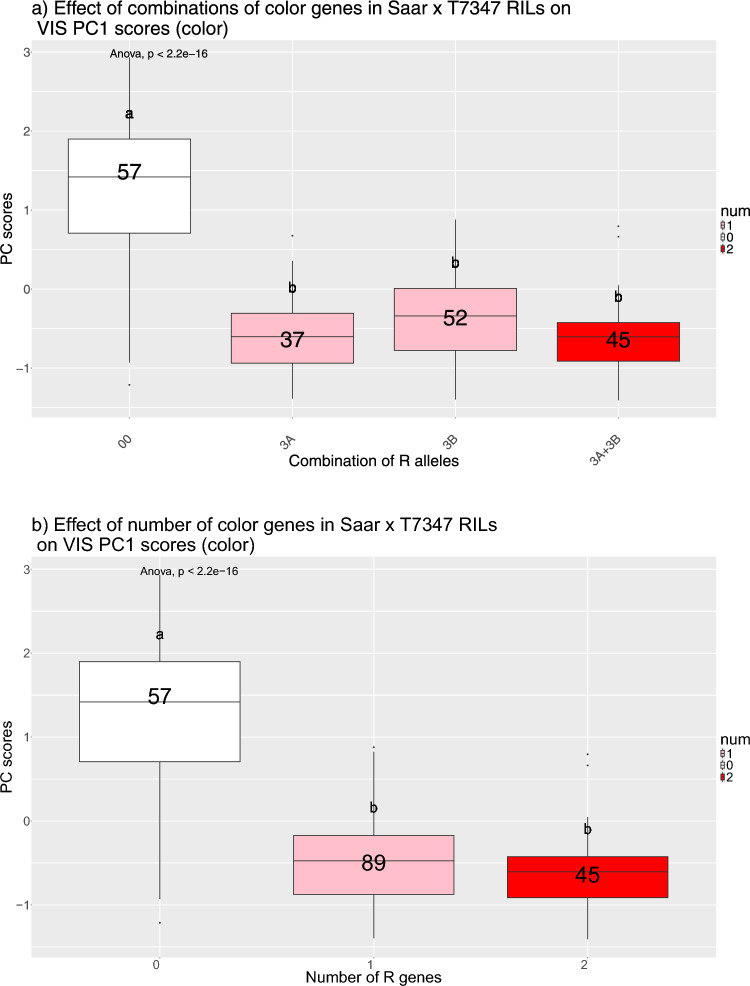
Boxplots showing the difference in PC1 scores for VIS reflectance, for groups of RILs with a) different allele combinations of the* R* genes on 3A and 3B, and b) whether they have 0, 1 or 2 of the *R* genes. The grouping is based on genotyping with the gene-specific KASP markers

**Fig. 5:**
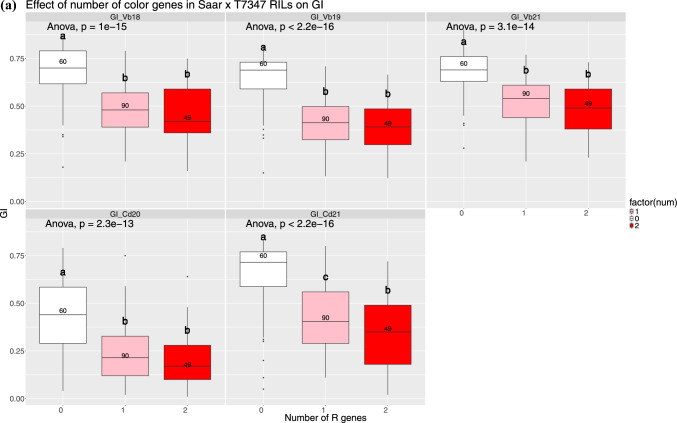

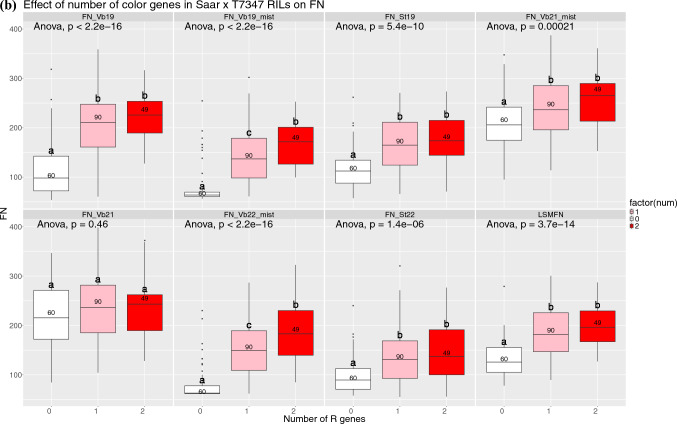
**a** Boxplots showing the effect on GI and **b** FN of having 0, 1 or 2 of the beneficial *TaMyb10*-alleles on 3AL and 3BL. Different letters indicate significant differences (based on Tukey’s HSD) between groups

ANOVA showed a significant effect between groups (Fig. 4), and Tukey’s HSD test was used to compare the groups. Between the effects of different combinations of the 3A and 3B alleles (0, 3A 3B or both) and between numbers of genes (0, 1 or 2), the only significant difference was detected between the groups having no color genes against one or two. Although a trend with decreasing PC1 scores with increasing number of *R* genes was seen, no significant differences were detected between the groups with one or two color genes. Since these results could indicate that the additive effect of having more than one color gene was not significant for grain color—at least not measured by NIR/VIS spectrometry, we decided to investigate the effect of the number of these genes on GI and FN (Fig. 5a, b).

Figure 5a indicates that in four out of five trials, there was no significant difference between having one or two color genes. In contrast, RILs with no color alleles were significantly less dormant than the other groups. However, the difference between groups with one or two color genes was significant in Chengdu, China, in 2021. For FN, a significant effect was observed in two trials with a high degree of sprouting damage (Figure S1 and Fig. 5b, the mist irrigated trials at Vollebekk in 2019 and 2022). In another trial (Vollebekk 2021) with minor sprouting damage, no significant difference was observed for FN between groups of RILs with 0, 1 or 2 *R* genes. In the remaining four trials and across (LSmeans), there was no significant additive effect from having two versus one *R* gene (Fig. 5a).

#### Allele stacking of PHS QTL in the RILs

**Fig. 6:**
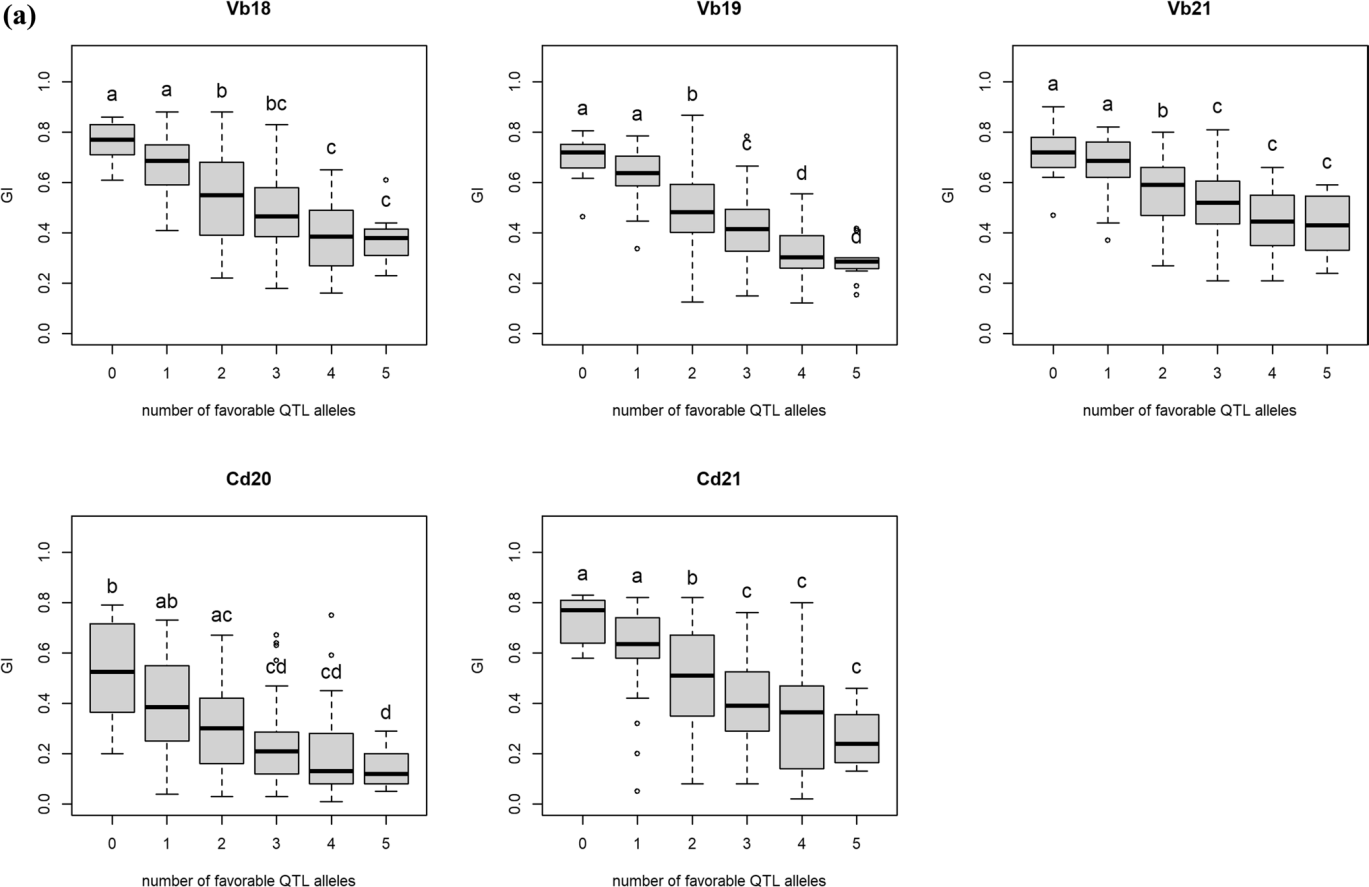

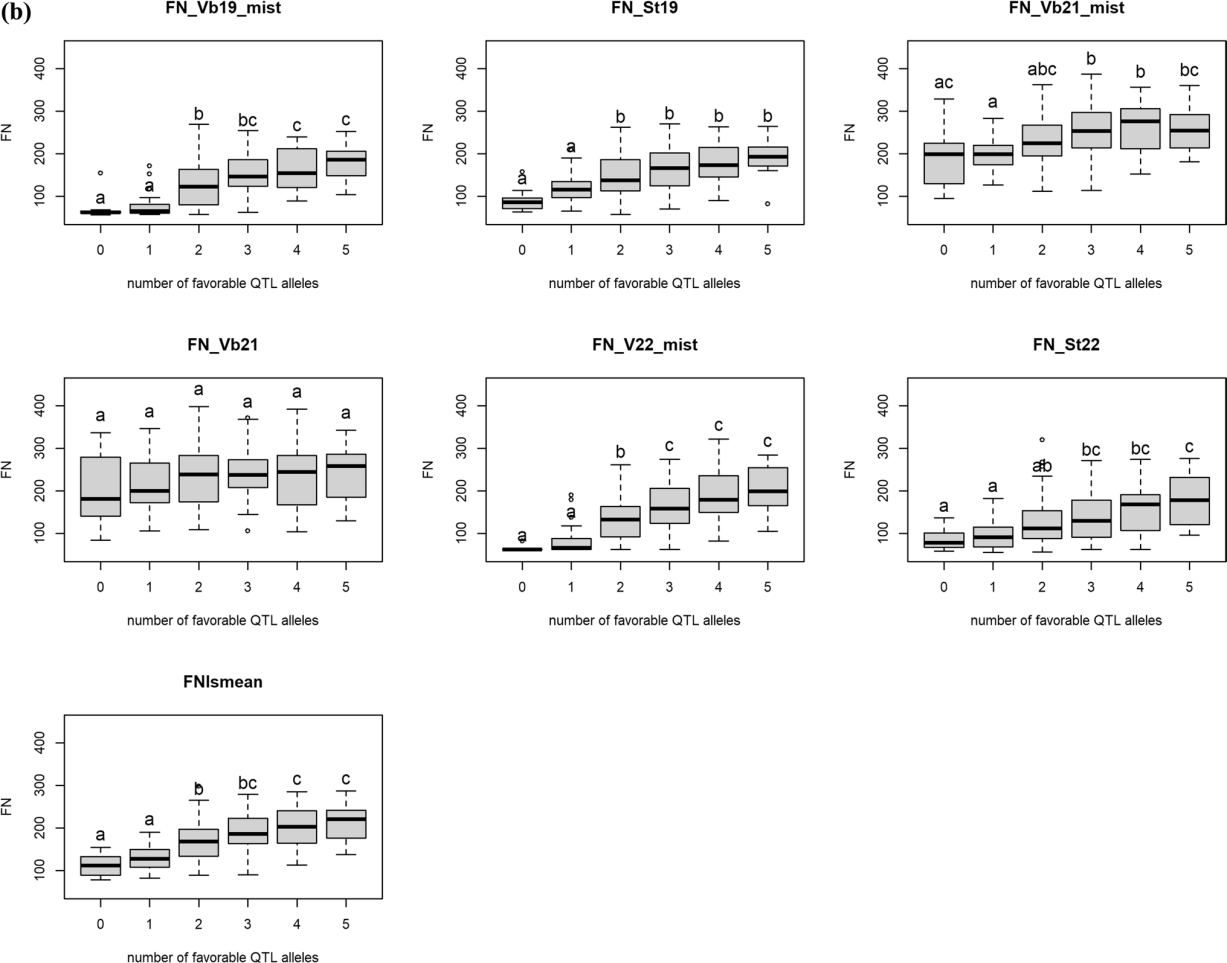
**a** Effect of number of PHS QTL alleles on GI in the individual trials. The letters above each box indicate significantly different mean GI by Tukey’s HSD test from groups not containing the same letter in the label, and **b** effect of number of the five PHS QTL on FN (LS means and individual years). The letters above each box indicate significantly different mean FN by Tukey’s HSD test from groups not containing the same letter in the label. Vb: Vollebekk, Norway; St: Staur, Norway

Allele stacking using markers from the five PHS QTL (1A, 3A, 3B, 7A and 7B) showed that two or more favorable QTL alleles were usually necessary to achieve significantly higher FN or lower GI than RILs with zero favorable QTL alleles (Fig. 6). On the other hand, the maximum number of favorable alleles to give a significant effect was four. In Vollebekk 2021, none of the PHS QTL summed up to significant differences on FN (Fig. 6b). In general, the effect of allele tacking was more consistent for GI than for FN, indicating that while dormancy is under stronger genetic control, FN is more influenced by environmental factors.

#### Marker validation in advanced breeding lines

The effects of markers linked with the identified GI and FN-related QTL were tested for a total of 6 trials with advanced breeding lines from Graminor AS, screened in different locations within south-eastern Norway (Staur, Bjørke, Rød, Vollebekk, Fig. S3 histograms, Table S1). The validation sets varied in composition and size between years (Table S2).

Markers linked to the 1A QTL (*QPHS.nmbu.SxT.1A.1*) were significant in Bjørke and Vollebekk in 2021 and Bjørke in 2022. Markers associated with the 3AL QTL had an effect in Staur 2019 and Bjørke 2022 at p < 0.1. 3BL was significant in Bjørke and Vollebekk 2021. The allele frequencies of the white color alleles on 3AL were low (< 0.05) in all the validation sets, while the beneficial allele on 3BL was minor but not rare with allele frequencies from 0.12 (2021) to 0.22 (2019).

*Rht-B1* (*QFN.nmbu.SxT.4B.1)* had a significant effect on FN in Bjørke and Vollebekk 2021 and Bjørke 2022, with the dwarfing allele contributing to higher FN, as expected.

Markers linked to the FN-specific 5AL QTL associated with awns in the biparental population, were significant in Staur 2019 and Bjørke 2022. However, only few lines per trial segregated for this region. The 7A markers were significant in all the trials except Rød 2019 and Bjørke 2020 (small set). The 7B QTL appeared to be fixed for the detrimental allele in the validation panels.

## Discussion

In this study, we screened a biparental population of 233 RILs for GI and FN data in a total of 12 trials in three different locations in Norway and China, between 2018 and 22. The location in Chengdu, China, was included to test the material under expected higher temperatures during the growing season. T7347 was selected as the resistant parent in this study due to its excellent level of dormancy even after higher temperatures during grain development. Over the past years, increased temperatures have been observed in many regions during the growing season. For this reason, it is relevant both for Norwegian wheat breeders but also for the global community to identify germplasm that is better fit to withstand PHS under elevated temperature regimes. In this study, we detected several QTL that were significant across locations in Norway and China.

Although FN data were only available from Vollebekk and Staur in Norway and GI data from Vollebekk and China were sampled in slightly different ways, the correlation between years/trials for GI and FN was significant and high. The higher correlation between DH and GI in China in 2020 could be explained by segregation for daylength sensitivity which was expressed in the short-day environment in Chengdu (data not shown, but the *Ppd-D1* locus on 2D was significant for DH in China). This effect on GI was probably strengthened by the sampling method used in the Chengdu trials, where spikes from all the RILs were sampled on the same date.

We defined stable PHS QTL as significant for more than one trial, affecting both GI and FN, and we identified five such QTL, on chromosome 1A, 3A, 3B, 7A and 7B with interval mapping (IM) in MapQTL6. Four of the beneficial alleles originated from T7347 and only one (3BL) from Saar. Additionally, two FN-specific QTL were identified, where the beneficial allele on 4B linked to the *Rht-B1* semi-dwarfing gene was contributed by Saar, and the one on 5AL (linked to awnless phenotype) was contributed by T7347. MQM mapping using markers identified with IM near or at the peak of the QTL as cofactors, helped to pinpoint the peak regions, but with this analysis the QTL on 7A could only be detected above the significance threshold of LOD 3 for GI.

The QTL on 1A was located close to the GA20-oxidase gene *TaGA20ox4-1A* (Izydorczyk et al. 2018), which is involved in the regulation of the level of bioactive GA in plant tissues (Yamaguchi 2008)*.*The QTL on 3AL and 3BL overlapped with the known locations of the R genes (*Myb10*- transcription factors). Subsequent genotyping with gene-specific KASP markers for *TaMyb10-A1* and *TaMyb10-B1* of the parents and a subset of the RILs (199 lines) and QTL mapping of color based on NIR/VIS analysis confirmed that the population segregated for these genes. The lines were monomorphic for the null allele for *TaMyb10-D1*. However, the regions also covered the *Vp1* loci on 3A and 3B. The 3A and 3B QTL were significant for all the GI trials in both Norway and China, but less consistent for FN.

The QTL on 7AL seemed to be novel. One interesting candidate gene close to the most significant markers was a dual specificity phosphatase (*TraesCS7A02G533600*), a subfamily of proteins that can be involved in regulation of ABA accumulation and ABA signaling (Ghelis et al. 2008; Monroe-Augustus et al. 2003)*.*

The QTL on 7B, located at around 221 Mbp, overlapped with a sprouting index-QTL (*QSi.crc-7B*) described by Cabral et al. (2014), who suggested abscisic acid responsive elements (*ABF2, ABF3*), located within orthologous regions from *Brachypodium* (*Bradi1*) and rice (*Os06*) to the QTL on 7B, as potential candidate genes. Based on the improved wheat genome resources available today, we suggest that another gene to investigate further is a *BHLH094*-like transcription factor (*TraesCS7B02G152800*). This type of transcription factor is known to be pleiotropic grain color genes in rice (Furukawa et al. 2007; Sweeney et al. 2006) and is a part of the *MYB-bHLH-WD4O* complex of genes regulating proanthocyanidin synthesis (Himi et al. 2023). However, the *bHLH* gene involved in this complex, regulating grain color in wheat, is not cloned yet. The QTL did not coincide with the 7B LMA locus which is located around 628 Mbp on the long arm of the chromosome (Derkx et al. 2021).

Two QTL with significant effect on FN, but not on GI, were identified on chromosomes 4B (from Saar) and 5AL (from T7347). The QTL on 4B was linked to the semi-dwarfing gene *Rht-B1*, which encodes a DELLA protein interacting with GA. Semi-dwarfs caused by mutant alleles of this gene are GA insensitive and known to have a pleiotropic effect on falling number and reduced α-amylase activity, as well as dormancy, depending on genetic background and environmental conditions (Derkx and Mvra, 2020; Gooding et al. 2012; Mares andMvra, 2014). The peak marker *RAC875_c48885_142* identified in most trials for the QTL on 5AL was located close to the *Tipped 1* (*B1*) locus (DeWitt et al. 2020; McIntosh et al. 2014) and 10 cM from a highly diagnostic marker for awn presence/absence in the NIAB MAGIC population (MacKay et al. 2014). However, only the peak marker on 5A for the FN trial in Staur 2019 mapped to the exact same cM position as *BobWhite_c8266_227.* In addition to a suspected negative effect of awns on PHS in humid, temperate climates by accumulating more dew and increasing the humidity within the spike, this gene has recently been characterized as a zinc finger transcription factor (DeWitt et al. 2020; Huang et al. 2020) regulating plant hormone pathways.

However, as the QTL regions span hundreds of genes, we can only speculate about the candidate genes underlying them. Transcriptome studies of dormant and non-dormant lines from the population, followed by functional validation experiments, may further illuminate these mechanisms.

The population was phenotyped for color with NIR/VIS spectroscopy to investigate the relationship between *R* gene dosage and grain color. One hundred and ninety-nine RILs and the parents were genotyped using gene-specific KASP markers for the *R* genes. Significant differences in color were only found between lines with zero and one or two *R* alleles, but not between one and two. Although we cannot exclude insufficient sensitivity of the phenotyping method, a similar, limited additive effect of the *R* gene dosage was observed for GI and FN. Exceptions were observed for FN in the mist irrigated trials in Vollebekk 2019 and 2022 (Fig. 5B) where a significant difference was also observed between lines with one and two genes. Significant differences were also seen for GI in Chengdu 2021, but interestingly not for the other four GI trials. Our results seem to align with Bassoi and Flintham (2005), where the largest differences in color and dormancy also were observed between lines with 0 and lines with red color, and the effect on dormancy of *R* gene dosage varied between trials.

Environmental factors including rain and pathogens might influence their relative importance, as well as confound the color intensity. Thus, it might still be beneficial to stack two or more color genes. The moderate size of the RIL population, limited number of recombination events and the quantitative nature of resistance to PHS might, however, have confounded the effects of R genes with beneficial alleles for other QTL. For this reason, the effect of stacking more than one R gene may have a larger effect in different germplasms.

Stacking up to four beneficial PHS QTL alleles had significant effect on both GI and FN, and it was usually necessary to have more than one beneficial QTL allele to achieve a significant difference from having zero beneficial QTL alleles. The effects of allele stacking were relatively stable across the years, particularly for GI (Fig. 5A). For FN, the most noticeable exception was Vollebekk 2021 where none of the PHS QTL added up to a significant difference from 0. The QTL analysis for this trial also showed that the FN-specific QTL on 4B and 5AL were the only significant QTL, indicating that most of the variation in FN was due to other factors than dormancy and PHS in this trial.

QTL identified in wide, biparental crosses may not be relevant in modern breeding material. We tested the effect of markers in the PHS and FN QTL regions (1A, 3A, 3B, 4B, 5A, 7A and 7B) on FN in six trials with validation panels consisting of modern varieties and advanced breeding lines from Graminor (described earlier). Markers linked to the QTL on 1A, 3AL, 3BL, 4B, 5AL and 7A had significant effects in the breeding material in more than one trial. Based on allele frequencies in the validation panels, the QTL on 3B, 7A and 7B hold the largest potential for PHS improvement in the Norwegian spring wheat breeding program, while the beneficial alleles on 1A and 3AL were very common (> 95% of the lines harboring the beneficial alleles). The detrimental allele for the QTL on 7B seemed to be fixed in the breeding material, so introgression of the beneficial allele is expected to improve the level of PHS resistance in the germplasm.

Markers linked to the 3AL QTL from T7347, associated with red grain color, had a significant effect in Staur 2019 and Bjørke 2022. 3BL was significant in Bjørke and Vollebekk 2021. Interestingly, the effect of markers linked to these loci in the validation panels was only significant for either markers on 3A, or on 3B, per trial. Since these populations varied in composition between years, it is difficult to conclude whether this was a coincidence or could be explained otherwise. As the allele frequency of the beneficial 3BL QTL from Saar was relatively low (up to 0.22) in the validation panels, there is a potential for allele enrichment for this QTL. However, the extent of additive effects of stacking more than one color gene should be investigated, as the populations already were almost fixed for the *R* gene on 3AL.

The 7A markers were significant in all the trials except Rød 2019 and Bjørke 2020 (small set), and both PHS resistance and susceptibility alleles are common in the advanced breeding lines. There is a potential to increase PHS resistance by allele enrichment for this QTL.

While the Ta*MFT-A1* gene has been identified as a regulator of temperature response to dormancy (Nakamura et al. 2011), we did not identify QTL on the short arm of 3A or other known *MFT* loci in our study. The explanation for T7347’s superior dormancy level even compared to today’s market varieties and breeding lines, may be found by combining at least one of the grain color genes with the other QTL on 1A, 7A and 7B. Ideally, this could provide improved temperature-stable dormancy on top of *MFT* which is often preferred as a dormancy source (Nakamura et al. 2011). T7347 or RILs with good allele combinations and better agronomical performance than the parent could potentially be used as donor parents. As the population was tested in Chengdu, China, one possibility is to use RILs that showed a comparably better dormancy after being grown under these warmer conditions. Seeds from the population can be shared freely. In the RILs, the grain color genes on 3AL and 3BL seemed to be the most important contributors to dormancy and higher FN in almost all the tested environments (Table 2). However, based on the marker validation analysis using the advanced breeding line panels, only the QTL on 7B is currently absent from the Norwegian spring wheat breeding germplasm.

*Rht-B1* had a significant effect on FN in Bjørke and Vollebekk 2021—when it was also significant in SxT—and Bjørke 2022, with the dwarfing allele contributing to higher FN, as expected.

Markers linked to the FN-specific 5AL QTL associated with awns in the biparental population, were significant in the advanced breeding lines in the marker validation trials in Staur 2019 and Bjørke 2022 (Table 3). Interestingly, the breeding lines were all awnless, but the QTL were associated with a haplotype close to, but distal (from approximately 704.5 Mbp) to the *Tipped 1* awn locus. However, only few breeding lines per trial segregated for this region. Since spike morphology including awns has been linked to PHS in humid, temperate climates, it could be interesting to further disentangle the awn phenotype and the distal haplotype to confirm whether there are two closely linked, but independent loci affecting FN.

**Table 3:** For trials with significant differences between genotypes segregating for a QTL: mean FN in groups of breeding lines with either the Saar or T7347 (in parenthesis) allele in the Graminor validation panels

QTL	Staur 2019	Bjørke 2021	Vollebekk 2021	Bjørke 2022
*QPHS.nmbu.SxT.1A.1*		319.7(345.2)	306.2(337.4)	278.7(309.7)
*QPHS.nmbu.SxT.3AL.1*	162.1(207.6)			210.2(305.6)
*QPHS.nmbu.SxT.3BL.1*		364.8(334.7)	360.1/(324.1)	
*QFN.nmbu.SxT.4B.1*		370.6(327.1)	365.3(315.8)	329.6(294.3)
*QFN.nmbu.SxT.5A.1*	137.9(210.3)			234.2(307.6)
*QPHS.nmbu.SxT.7AL.1*	193.9(224.2)	326.4(340.1)	310.3(353.8)	292.3(315.6)

While PHS is a complex trait, we could identify five stable QTL significant for both GI and FN across most tested environments. Four of these, as well as the two FN-specific QTL, could be validated in the advanced breeding panels, proving that the cross based on a relatively old Norwegian breeding line (T7347) and an exotic line (Saar) from CIMMYT harbored relevant genetic variation, and that there is a potential to enrich the frequency of beneficial alleles for the investigated QTL in the breeding material, to further improve PHS resistance.

Further work includes transcriptome analysis of dormant and non-dormant lines from the population to increase the understanding of genetic mechanisms underlying the QTL.

T7347 is particularly interesting due to its maintenance of dormancy under high temperatures. Although the combined dataset is large and sampled from different environments, we could not conclude about the effects of individual QTL under different temperatures. We only had FN data from Norway, and we could not exclude plant developmental stage as a reason for differences between QTL for GI in Norway and China due to different sampling strategies. RILs that retain dormancy better than others under warmer growing conditions like in China would be particularly interesting to investigate further or use as donors of improved PHS. Thus, it would be useful to investigate in a more systematic way over a larger number of environments whether we can validate QTL contributing to sufficient levels of dormancy also under conditions with increased temperature.

## Supplementary Information

Below is the link to the electronic supplementary material.

Figure S1. Histograms of phenotypic distribution among the 233 SxT RILs for agronomical traits, germination index (GI) and falling number (FN). DH=Days from sowing to heading, DM=Days from sowing to physiological maturity and PH=Plant height. “Mist” indicates trials where mist irrigation was applied to induce PHS after maturation in seasons with low rainfall. Trial location abbreviations: Vb= Vollebekk, Norway, St = Staur, Norway, and Cd = Chengdu, China (PDF 12 kb)

Figure S2. PCA plot of PC1 and PC2 of SNV transformed VIS data for the SaarxT7347 RIL population (233 lines) and parents. PC1 explained > 92% of the phenotypic variation. (PDF 18 kb)

Figure S3. Histogram of distribution of RILs and parents in the Saar x T7347 population, based on the first-principal component (PC1) of VIS data calculated from all wavelengths (400–800 nm) used for grain color measurements. The values of the parents are marked with a red and blue “x” for Saar and T7347, respectively. (PDF 4 kb)

Figure S4. Histograms of phenotypic distribution of falling number (FN) in Graminor validation populations (advanced breeding lines) 2019–22. (TIFF 12802 kb)

Supplementary file5 (XLSX 7256 kb)

Supplementary file6 (DOCX 13 kb)

## Data Availability

Data generated and analyzed for this study will be uploaded as supporting material before publication. Sequences for the gene-specific KASP primers can be provided upon request from Dr. Gina Brown-Guedira at USDA-ARS, North Carolina.
