# Supplementary figures and images for: Quantitative trait loci mapping of resistance to pre-harvest sprouting in the Norwegian spring wheat breeding line T7347
Source: Theor Appl Genet. 2025 Jun 24;138(7):159. doi: 10.1007/s00122-025-04943-7 (PMC12185635; doi:10.1007/s00122-025-04943-7)

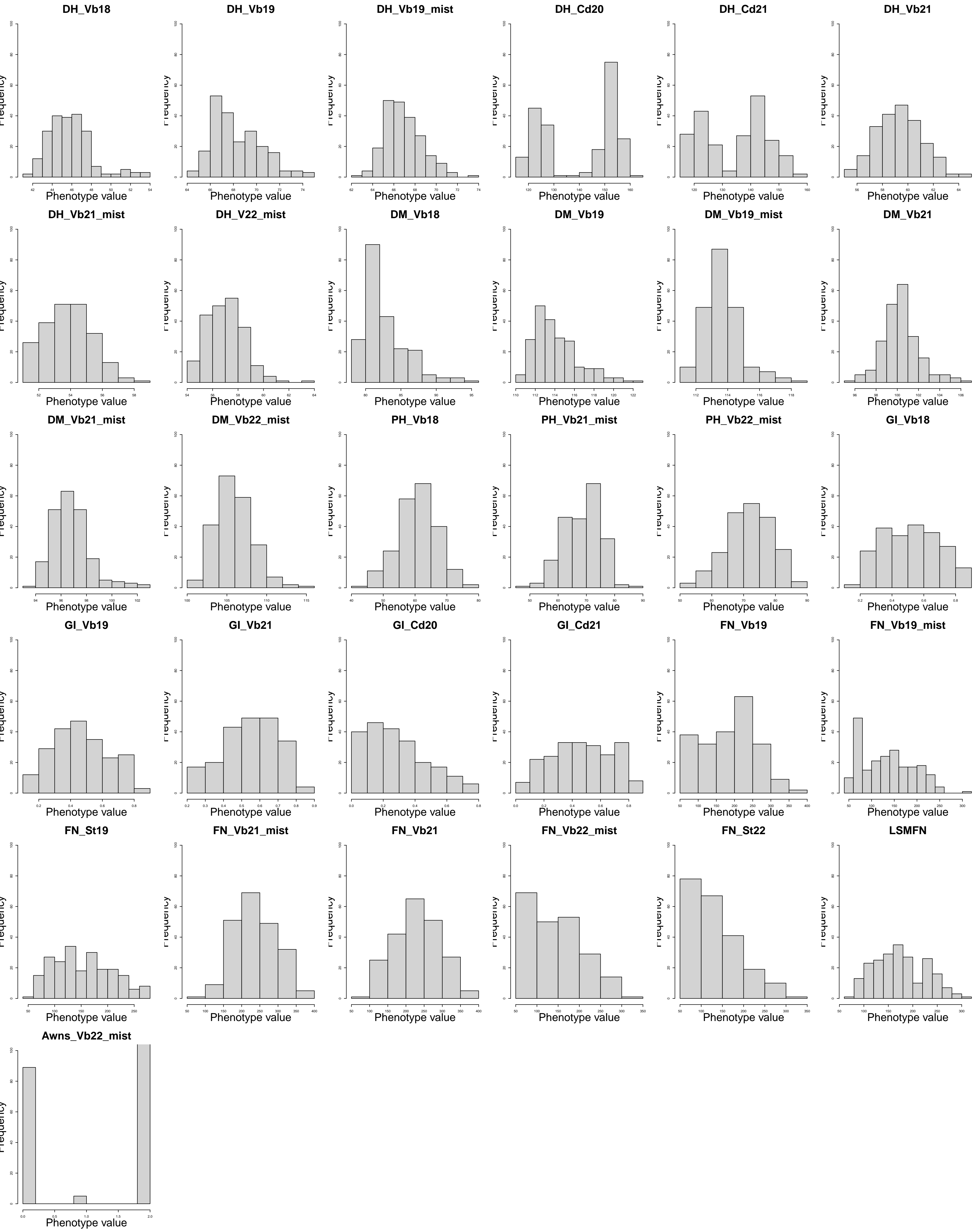

Supplement: Supplementary file 1 — Figure S1. Histograms of phenotypic distribution among the 233 SxT RILs for agronomical traits, germination index (GI) and falling number (FN). DH=Days from sowing to heading, DM=Days from sowing to physiological maturity and PH=Plant height. “Mist” indicates trials where mist irrigation was applied to induce PHS after maturation in seasons with low rainfall. Trial location abbreviations: Vb= Vollebekk, Norway, St = Staur, Norway, and Cd = Chengdu, China (PDF 12 kb) [file 122_2025_4943_MOESM1_ESM.pdf]

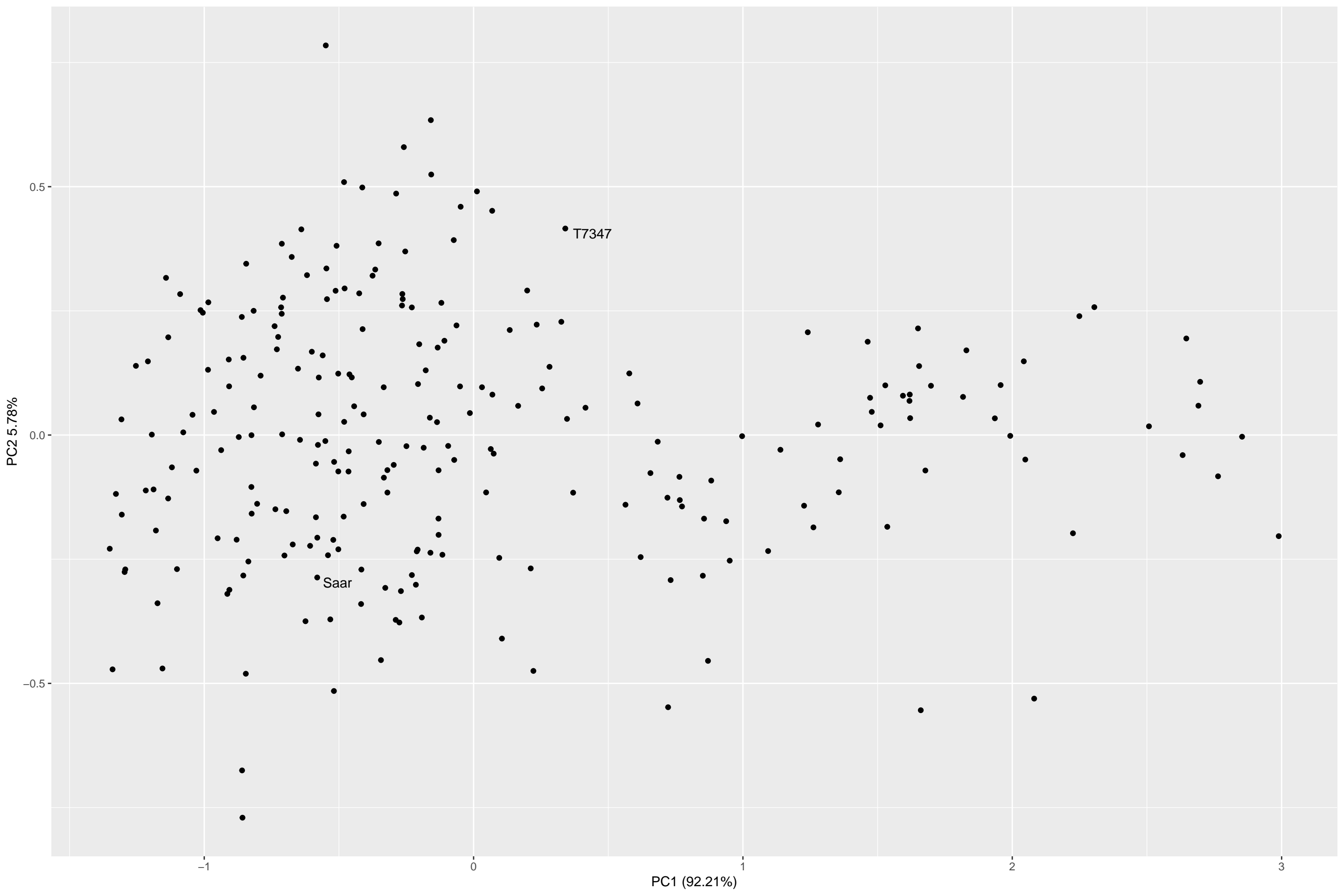

Supplement: Supplementary file 2 — Figure S2. PCA plot of PC1 and PC2 of SNV transformed VIS data for the SaarxT7347 RIL population (233 lines) and parents. PC1 explained > 92% of the phenotypic variation. (PDF 18 kb) [file 122_2025_4943_MOESM2_ESM.pdf]

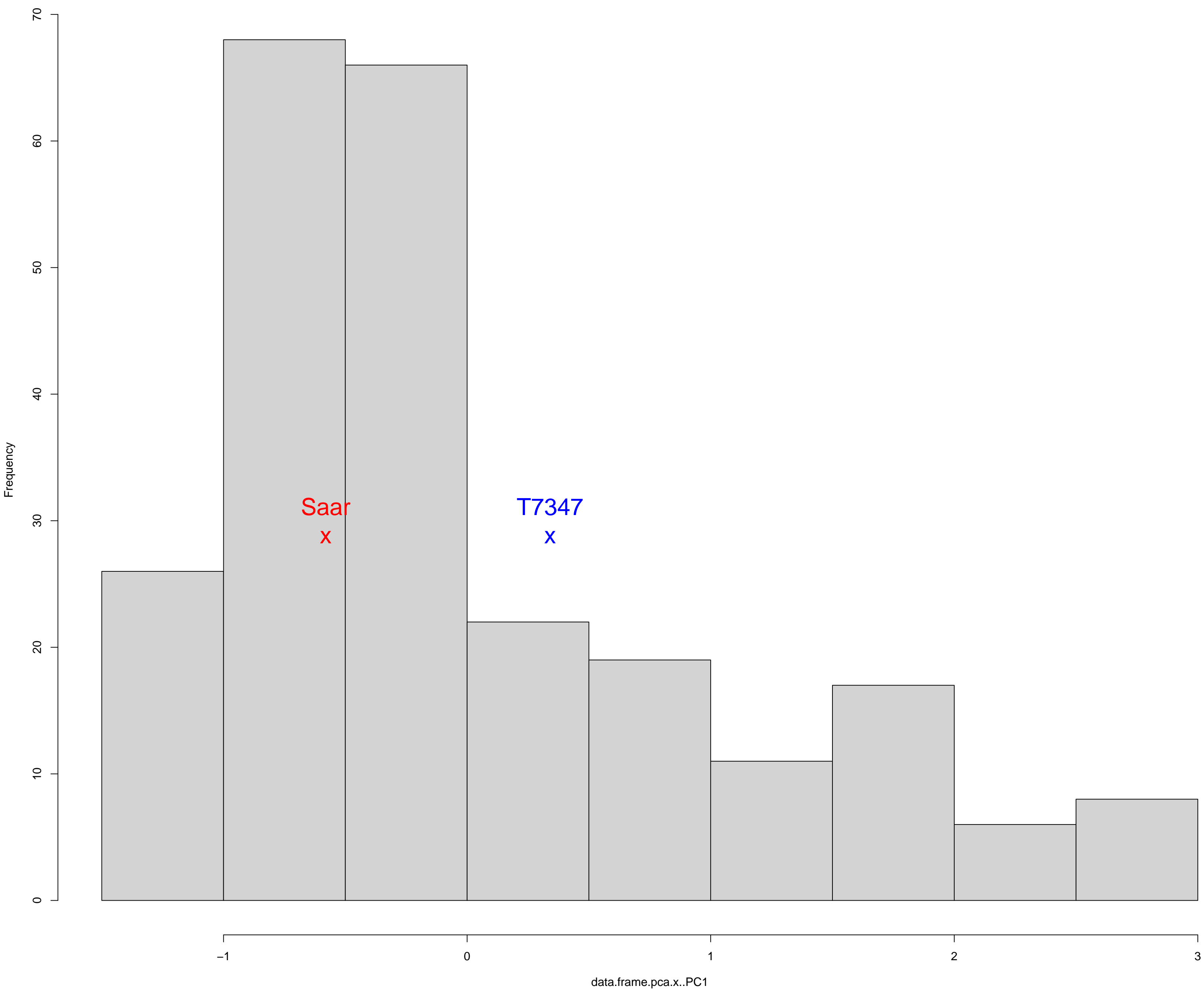

Supplement: Supplementary file 3 — Figure S3. Histogram of distribution of RILs and parents in the Saar x T7347 population, based on the first-principal component (PC1) of VIS data calculated from all wavelengths (400–800 nm) used for grain color measurements. The values of the parents are marked with a red and blue “x” for Saar and T7347, respectively. (PDF 4 kb) [file 122_2025_4943_MOESM3_ESM.pdf]

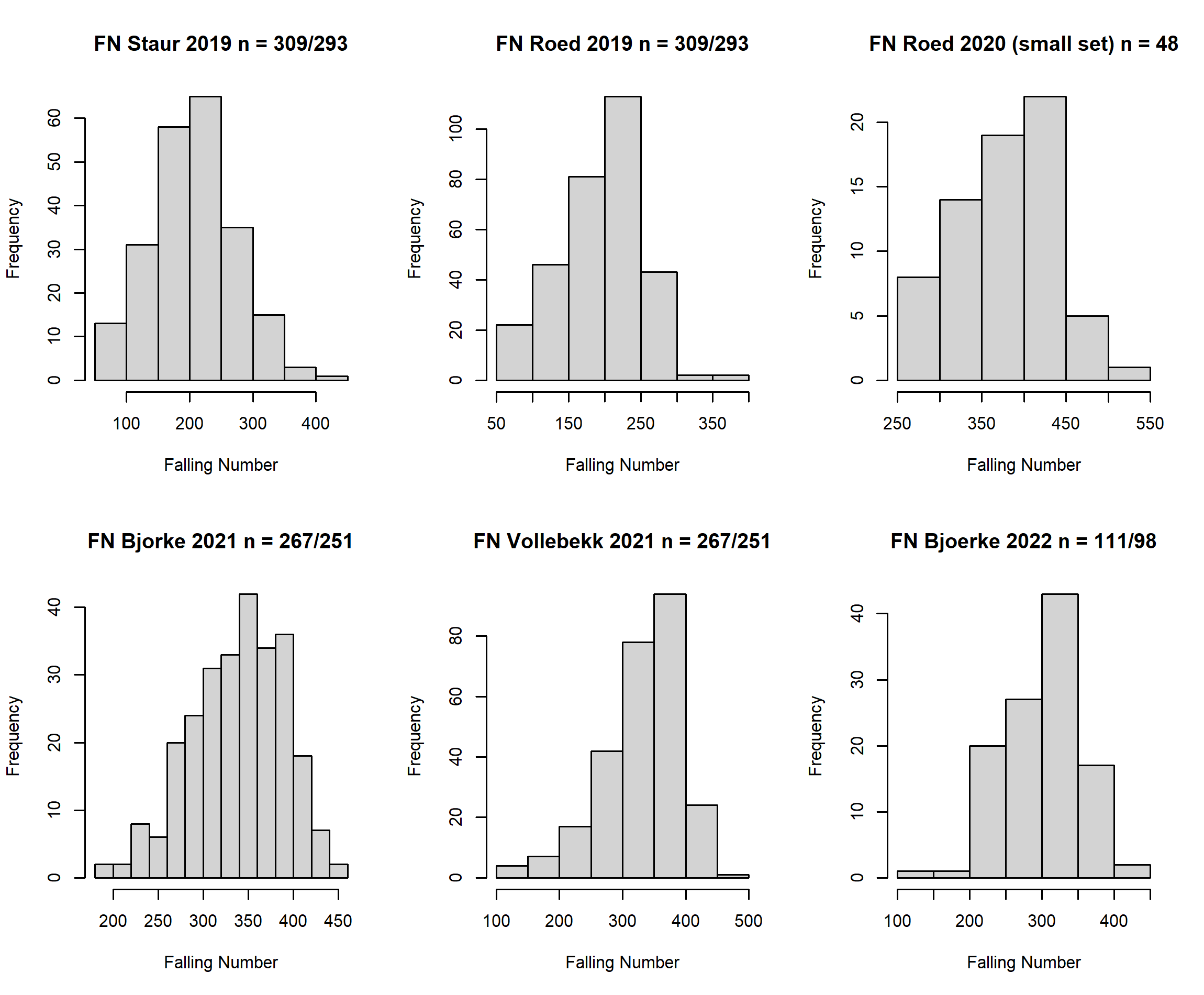

Supplement: Supplementary file 4 — Figure S4. Histograms of phenotypic distribution of falling number (FN) in Graminor validation populations (advanced breeding lines) 2019–22. (TIFF 12802 kb) [file 122_2025_4943_MOESM4_ESM.tiff]
