# Supplementary material for: Quantitative trait loci mapping of resistance to pre-harvest sprouting in the Norwegian spring wheat breeding line T7347
Source: Theor Appl Genet. 2025 Jun 24;138(7):159. doi: 10.1007/s00122-025-04943-7 (PMC12185635; doi:10.1007/s00122-025-04943-7)
Supplement: Supplementary file 6 — Supplementary file6 (DOCX 13 kb) [file 122_2025_4943_MOESM6_ESM.docx]

Supplementary tables

| Table S1 Markers used for allele stacking of the SxT RILs. | |
| --- | --- |
| QTL name | Marker |
| *QPHS.nmbu.SxT.1A.1* | *AX-158537396* |
| *QPHS.nmbu.SxT.3AL.1* | *TaMyb10-A1* |
| *QPHS.nmbu.SxT.3BL.1* | *TaMyb10-B1* |
| *QPHS.nmbu.SxT.7AL.1* | *Ku_c19745_892* |
| *QPHS.nmbu.SxT.7B.1* | *Tdurum_contig25631_143* |

| **Table S2** Number of advanced breeding lines (along the diagonal) in validation trials (2019-2022) and overlap of genotyped lines between years, in green) | | | | |
| --- | --- | --- | --- | --- |
|  | **2019** | **2020** | **2021** | **2022** |
| **2019** | 293 |  |  |  |
| **2020** | 47 | 48 |  |  |
| **2021** | 61 | 25 | 251 |  |
| **2022** | 26 | 12 | 67 | 98 |

| **Table S3** Markers used for marker validation in the advanced breeding line. Since these lines were not genotyped with gene specific KASP markers on 3A and 3B, we used closely linked markers from the 25 K SNP chip, listed here. | | | | |
| --- | --- | --- | --- | --- |
| QTL name | marker | cM position in SxT | Phys pos Mbp | Comment |
| *QPHS.nmbu.SxT.1A.1* | *AX-158537396* | 147.670 | 460.33 |  |
| *QPHS.nmbu.SxT.3AL.1* | *AX-94898331* | 121.423 | 708.23 |  |
| *QPHS.nmbu.SxT.3BL.1* | IAAV6088 | 234.704 | 759.16 |  |
| *QFN.nmbu.SxT.4B.1* | *TG0010a* | 144.18 | 30.86 |  |
| *QFN.nmbu.SxT.5A.1* | *RAC875_c48885_142* | 103.492 | 706.541* | *Best blast hit |
| *QPHS.nmbu.SxT.7AL.1* | *Ku_c19745_892* |  |  |  |
